# Utilization of a Biodegradable Mulch Sheet Produced from Poly(Lactic Acid)/Ecoflex^®^/Modified Starch in Mandarin Orange Groves

**DOI:** 10.3390/ijms10083599

**Published:** 2009-08-17

**Authors:** Yuya Tachibana, Takuya Maeda, Osamu Ito, Yasukatsu Maeda, Masao Kunioka

**Affiliations:** 1 Industrial Technology Center of Wakayama Prefecture, 60, Ogura, Wakayama, 649-6261, Japan; E-Mails: yuya-tachibana@aist.go.jp (Y.T.); o-itoh@wakayama-kg.go.jp (O.I.); ymaeda@wakayama-kg.go.jp (Y.M.); 2 National Institute of Advanced Industrial Science and Technology, 1-1-1, Higashi, Tsukuba, Ibaraki, 305-8565, Japan; E-Mail: m.kunioka@aist.go.jp (M.K.)

**Keywords:** poly(lactic acid), modified starch, mulch sheet, mandarin orange, biodegradable materials, biomass carbon ratio

## Abstract

We have developed a mulch sheet made by inflation molding of PLA, Ecoflex^®^ and modified starch, which all have different biodegradabilities. A field test of use as an agricultural mulch sheet for mandarin oranges was carried out over two years. The mechanical properties of the mulch sheet were weakened with time during the field test, but the quality of the mandarin oranges increased, a result of the controlled degradation of the sheet. The most degradable modified starch degraded first, allowing control of the moisture on the soil. Accelerator mass spectroscopy was used for evaluation of the biomass carbon ratio. The biomass carbon ratio decreased by degradation of the biobased materials, PLA and modified starch in the mulch sheet.

## 1. Introduction

**Figure 1.**
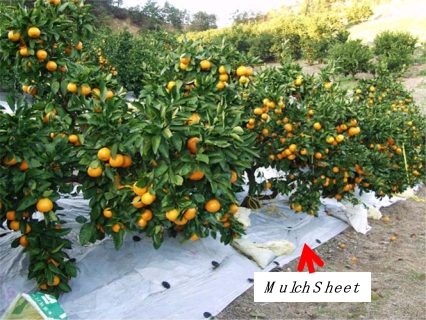
A mulch sheet in a mandarin grove.

A mulch sheet is an indispensable agricultural material for high-value added agricultural crops like sweet potatoes, strawberries, and oranges [1]. By using a mulch sheet on the soil, the temperature of the covered soil rises, the soil water content is controlled, weeds are reduced, and harmful insects are repelled. A white mulch sheet can reflect the solar light towards the downside of the agricultural crops, and the reflected light makes the crops grow faster and in the case of fruits, the quality of the crops increases due to the increased sweetness from the presence of more sucrose, which is produced by photosynthesis inside the fruit by the enhanced growth. Further, the need for agricultural labor decreases due to the decreased weeding and the presence of fewer harmful insects. Especially for growing mandarin oranges, which are one of the most popular fruits in Japan, the use of mulch sheets to cover the soil results in improved taste of the fruit and outside peel color (Figure 1) [2].

Usually, low-density polyethylene, high-density polyethylene or polyvinylchloride are used as the materials for manufacturing mulch sheets, although these materials cause some problems when used in an agricultural field. After use the mulch sheets produce a large amount of waste, and this requires additional labor and waste management and the disposal costs are very high. Moreover, the fragments of sheets broken during use remain in the soil, because these materials are not biodegradable. Biodegradable materials are gaining attention as a solution to these problems of mulch sheets. Nowadays, materials such as poly(lactic acid) (PLA), poly(butylene succinate), polycaprolactone, or poly(butylene adipate/terephtalate) (commercially supplied by BASF under the tradename Ecoflex^®^) are being adapted as biodegradable mulch sheets [3,4].

PLA produced from biorenewable resources, such as corn, has recently been gaining attention for sustainability reasons, because the term “biorenewable” refers to materials made from biomass with absorbed carbon dioxide from the atmosphere [5]. However, its most important property is its biodegradability.

Ecoflex^®^ is also commercially available as a biodegradable material. It has a poly(butylene adipate/terephtalate) chemical structure and it is made from oil-based compounds. Currently, it is used as a sheet, fiber, and modifier for plastics [6].

Starch is one of the most inexpensive and most available biobased and biodegradable materials, although it is hard to use as a plastic-like material as it lacks moldability and thermoplasticity. Some modified methods have been developed to confer moldability and thermoplasticity to starch. Thus, a starch is usually treated by hydrolysis and functionalization with a glycol, then the modified starch becomes moldable and thermoplastic, in addition to biodegradable [7]. Generally, modified starch is melted and mixed with other thermoplastics for use as a functional material [8].

PLA, Ecoflex^®^, and modified starch are all biodegradable materials, therefore, they could solve the problem of mulch sheet biodegradability. The most economical method of producing a sheet is by inflation molding, but an adequate melt flow rate of the neat resin is needed for this. It is difficult to mold PLA and/or modified starch due to their bad melt flow rates, so it is necessary to use a modifier such as Ecoflex^®^. Another interesting feature results from the use of three different materials, because their degradation rates in a mandarin orange grove are different. The most degradable material, the modified starch, degrades at the beginning soon after the soil is covered, then the water permeability increases and this has a good effect on the mandarin orange growth, so the orange fruit becomes sweeter due to the increasing levels of sweeteners such as sucrose that are formed.

**Figure 2.**
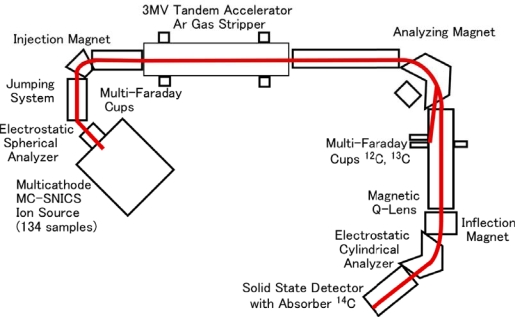
Outline of the accelerator mass spectroscopy (AMS) apparatus (size ca. 15 × 10 m, height 2 m) for determining the percentage of modern carbon (pMC) by the ratio of ^14^C/^12^C (^14^As) at the Institute of Accelerator Analysis, Ltd., Japan.

PLA and modified starch are biomass materials. When a product is used as a biobased material, it is important to measure the biomass carbon ratio. Recently, the accelerator mass spectroscopy (AMS) (Figure 2) measurement method for determining the biomass carbon ratio based on the American Society for Testing and Materials (ASTM) D6866 method entitled “Standard test methods for determining the biomass carbon ratio of solid, liquid, and gaseous samples using radiocarbon analysis” has become very important in the field of biomass plastics [9,10]. The AMS method has been used for the dating of archaeological and geological samples. In addition, the method can be applied to a mass balance study using a lower amount of ^14^C-labelled metabolic compounds for biological systems. AMS can measure a very small ^14^C concentrations and determine the ratio of the radiocarbon-14 to carbon-12 and 13. The ratio ^14^C/^12^C in biomass carbon is around 1 × 10^−12^. Fossil resources and materials are those that contain no ^14^C because all ^14^C atoms have a 5,730 year half-life time.

**Figure 3.**
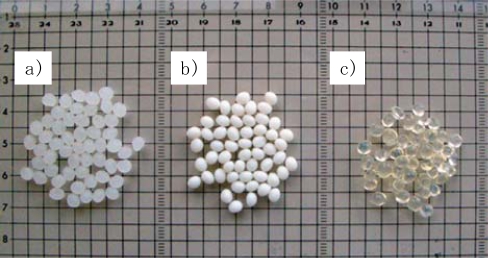
Pellet of a) PLA, b) Ecoflex, c) modified starch.

In this study, we molded mulch sheets consisting of PLA, Ecoflex^®^ and modified starch, which are commercially available and fully biodegradable polymers, and used them in a mandarin orange grove. Each type of resin is shown in Figure 3. Due to the differences in biodegradability, the properties of the mulch sheets gradually change, and the changes in the water permeability have a positive effect on the mandarin orange growth. The biomass carbon ratio and biodegradability were measured by the AMS method.

## 2. Results and Discussion

### 2.1. Inflation Molding

**Figure 4.**
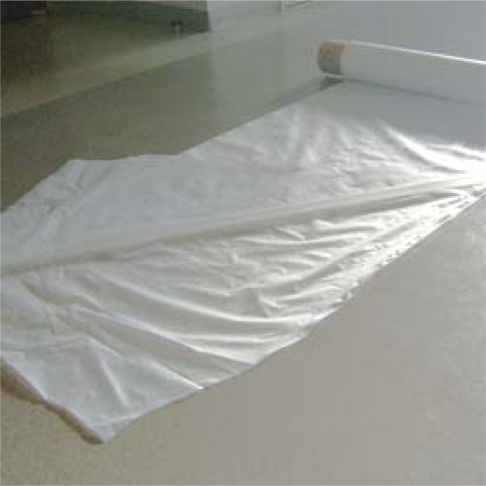
Double layered MS-3,6,1 produced by inflation molding from PLA/Ecoflex**^®^**/modified starch. The width is 1 m, the length is 100 m, and the thickness was 30 μm (2004 year), or 90 μm (2005 year) (double layered).

When a sheet is to be used for agricultural mulch, it is necessary to mold a thin and wide sheet. Inflation molding is one of most inexpensive and facile molding methods for manufacturing thin and wide sheets. For inflation molding, a suitable melt viscosity is required [11]. A change in the ratio of the resins can cause the viscosity to change. The ratio of the resins, PLA, Ecoflex^®^, and modified starch and the moldability are shown in Table 1. The sheet names denote the ratio of the resins in the mulch sheet; the first number is PLA, the second is Ecoflex^®^, and the last is modified starch. Although PLA, which was used in this study, could not be molded with my apparatus as a thin sheet (MS-10,0,0), PLA with Ecoflex^®^ could be molded, because Ecoflex^®^ has good properties for inflation molding. When adding modified starch, the sheet could not be molded. Finally, the sheet, including modified starch, could be molded when the ratio of PLA/Ecoflex^®^/modified starch is 3/6/1. It shows that Ecoflex is necessary for molding PLA and modified starch. All sheets were molded as tubular sheets and rolled up in double layered sheets, as shown in Figure 4. The width is 1 m, the length is 100 m, and the thickness of a single layer is 30 μm (2004 year), or 90 μm (2005 year). We used two sheets, MS-3,7,0 and MS-3,6,1, as the mulch sheet after the sheets were cut into a single layer.

### 2.2. Properties of Sheets

**Figure 5.**
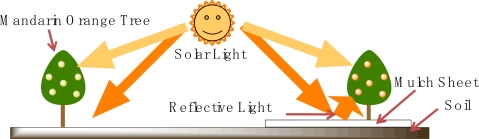
Mechanism of reflection of solar light using mulch sheets in mandarin orange groves.

The optical properties of a mulch sheet are important. The transmittance affects the soil, and the temperature increases due to the greenhouse effect. Solar light is necessary for the growth of plants, but the down side of a plant will not get sufficient light, so it is effective to illuminate the plant with another light source. Especially, in the case of mandarin oranges, the lighted fruit has a deep peel color and good fruit taste. The easiest method is utilization of reflected light from the soil as shown in Figure 5. Because the reflectance from the soil is generally low, a highly reflective sheet is laid on the soil.

The optical properties are shown in Table 2. The diffuse transmittance of MS-3,7,0 with a 30 μm thickness is due to the immiscible PLA domain. The mulch sheets transmit about half of the light of a 90 μm thickness. This is sufficient for the greenhouse effect on the soil. The diffuse reflectance of the mulch sheets is two times greater than the soil.

The gas permeabilities of oxygen, nitrogen, and moisture are shown in Table 3. These sheets have a gas barrier. If the sheet completely blocks the moisture, the root decays with the input of water from around the soil or dries out without it. So, moderate moisture permeability is needed as will be shown later.

### 2.3. Field Tests in Mandarin Orange Groves

**Figure 6.**
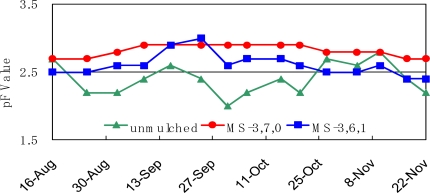
pF value of the soil in orange grove at Wakayama in 2004 after mulching on 1^st^ July.

A field test for growing mandarin oranges was carried out in 2004 and 2005. The sheets provided a 4 × 4 m mulch around the tree from 17^th^ July to 22^nd^ November in 2004 and from 7^st^ July to 7^th^ November in 2005. The moisture regain on the soil in 2004 is shown in Figure 6. The pF value, the negative logarithm of the capillary strength, was used as an indicator of the moisture regain on the soil. If the soil is dry, the capillary strength become strong and the pF value become high. MS-3,7,0 kept the pF value of the mulched soil higher than the unmulched soil for the entire growth period. On the other hand, MS-3,6,1 kept it higher than the unmulched soil from the early period to the medium period, however, the pF value is lower Therefore, it means that the soil mulched with MS-3,6,1 was kept dry for the early and medium periods of growth and moderately wet for the last period. It is supposed that the moisture permeability of MS-3,6,1 changed with time. Unfortunately, the moisture permeability of the used sheets could not be measured because the sheets became fragile.

**Figure 7.**
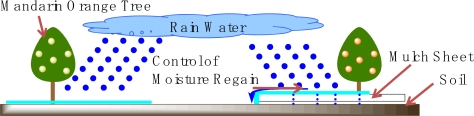
Control mechanism of moisture regain by soil using biodegradable mulch sheet.

It is supposed that the change was caused by the mechanism illustrated in Figure 7. In the early period, the rainwater drained along the surface of the mulch sheet, and the soil was kept dry. After the medium period, the rainwater could seep through the mulch sheet, because the mulch sheet degraded, and the soil was kept wetter than the unmulched soil. For the growth of mandarin oranges, dry soil is suitable for making a good tasting fruit. However, it is unsuitable to keep the soil very dry for the entire period [12]. Usually, the mulch drip method is adopted for the moisture control, however the cost of the apparatus is expensive [13]. Indeed, the change in the moisture uptake of soil in MS-3,6,1 represents the ideal condition.

**Figure 8.**
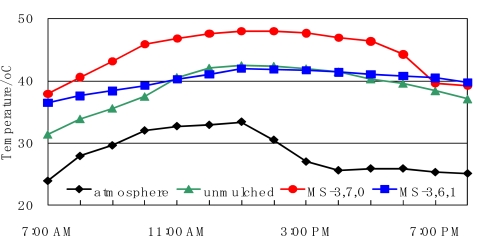
Temperature of the soil and atmosphere on 27 August, 2004.

The temperature changes of the soil on 27^th^ August, 2004, are shown in Figure 8. The temperatures of the mulched soil were higher than the unmulched soil. This shows that the mulch sheets produced a greenhouse effect. MS-3,6,1 kept the soil temperature warm and stable in the vicinity of 40 °C. However, MS-3,7,0 kept it high at around 50 °C. It is believed that the difference was caused by the change in the moisture permeability, and the change was caused by the difference in degradability. If the temperature of the soil is too high, it causes serious damage to the tree.

Generally the Brix degree, acid value, and peel color are used as indicators for the evaluation of the quality of mandarin oranges. The Brix degree indicates the dissolved sugar mass ratio in crush fruit and converted to a sucrose concentration value. Usually, a mandarin orange having over a 10 Brix% provides a good taste for any consumer. In addition to the Brix degree, the acid value is an important factor for taste. The acid value is measured by neutralization titration and converted to a ratio of citric acid. Some consumers prefer a low acid value (under 1%). However, other consumers prefer a high acid value (over 1%), if the Brix degree of the mandarin orange is over 10% [14]. Therefore, an adequate acid value and Brix degree are necessary to provide a preference. Peer color is also an important standard as a visual effect during it sale in Japan. Because the Japanese consumer prefers a dark orange color, the *a* value is an evaluation indicator for a high value-added mandarin orange.

**Figure 9.**
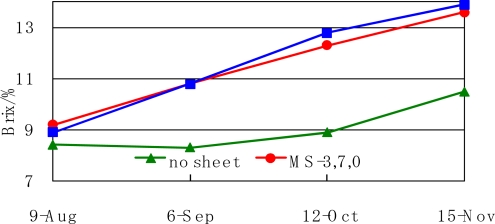
Variation in Brix degree of mandarin orange fruit after mulching on 1 August 2005.

The change in the average Brix degree of 10 fruits harvested from one trees versus time in 2005 is shown in Figure 9. In the early period, the Brix degrees of mulched and unmulched oranges are almost the same value. After one month, the Brix degrees of the mulched oranges are higher than the unmulched oranges. This good effect was caused by the control of the moisture regain of the soil as explained in Figure 6.

The evaluation of the mandarin orange is shown in Table 4. This evaluation was the average of 30 fruits harvested from four trees. The Brix degrees of the mulched fruits are higher than the unmulched fruits, especially the mulched fruit with MS-3,6,1 is the highest. No effect on the acid value was observed in 2004, but it clearly appeared in 2005. It seems that the effect on the Brix degree and acid value was caused by the control of the moisture regain by the soil. The peel color also became good in both years due to the effect of the reflected light by the mulched sheets. The difference in the effect of the mulched sheets by growth year was caused by the soil grade difference. In 2004, the field test was carried out on a flat grade, but in 2005 on a terraced grade. The terraced grade is more suitable for the growth of mandarin oranges, so the mulched sheets had a striking effect. It shows that a high value-added mandarin orange, having a good taste and fine visual effect, can be harvested using mulch sheets.

### 2.4. Degradation of Mulch Sheets

**Figure 10.**
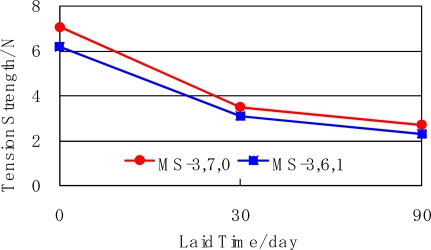
Tension strength of mulch sheets laid on mandarin orange grove.

**Figure 11.**
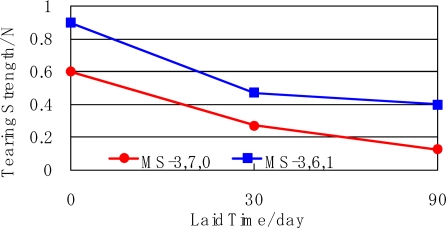
Tearing strength of mulch sheets laid on mandarin orange grove.

The physical properties of the mulch sheets, which were used from 1^st^ August to 26^th^ November in 2005 were measured before and after the field test. The tension strength and tearing strength along the traverse direction is shown in Figures 10 and 11. The strength decreased with time. It shows that the mulch sheets biodegraded.

**Figure 12.**
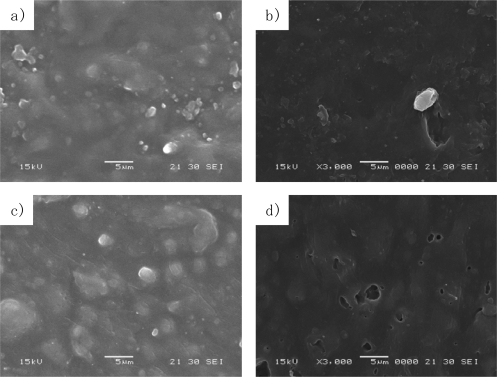
SEM images of (a) unused MS-3,7,0, (b) used MS-3,7,0, (c) unused MS-3,6,1, (d) used MS-3,6,1. Used sheets were mulched for 4 months.

Scanning electron microscopy (SEM), infrared spectroscopy (IR), and gel permeation chromatography (GPC) were completed to confirm the biodegradation of the mulch sheets. The SEM images are shown in Figure 12. As PLA used in this study is not fully compatible with Ecoflex^®^, the PLA domain remains as a rough surface (Figure 12a). Part of the domain was stripped after the field test as shown in Figure 12b. It is believed that Ecoflex^®^ as the matrix resin of MS-3,7,0 degraded, and the PLA domain was fragile and became stripped. Adding modified starch, a rougher surface appeared (Figure 12c). After the field test, some voids were formed as shown in Figure 12d. The voids were observed only in MS-3,6,1, then it seems that modified starch formed them as a result of the degradation.

**Figure 13.**
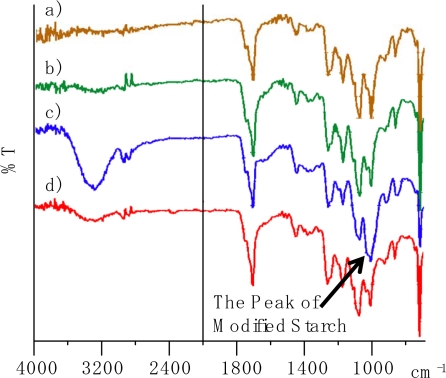
IR spectra of (a) unused MS-3,7,0, (b) used MS-3,7,0, (c) unused MS-3,6,1, (d) used MS-3,6,1. Used sheets were mulched for four months. The CO_2_ peak was omitted for clarify.

The IR spectra are shown in Figure 13. The peak of MS-3,7,0 appeared as a mixed peak of PLA and Ecoflex**^®^** (Figure 13a). The peak of MS-3,6,1 appeared as a mixed peak of PLA, Ecoflex**^®^** and modified starch, and the peak of modified starch appeared around 1,000 cm^−1^. It seems that there was no change in the peaks of MS-3,7,0 that could be distinguished by the IR spectra after the field test. Even if PLA and Ecoflex**^®^** degraded in the field test, the degradation was caused by the scission of the molecular chain and showed little change in the IR spectrum [15].

On the other hand, the IR spectra of MS-3,6,1 showed that the peak of modified starch disappeared after the field test, and there was no change in the peaks of PLA and Ecoflex^®^ in the IR spectra. It appeared that modified starch was lost during field test due to the degradation of modified starch. The degradation of modified starch is faster than PLA and Ecoflex^®^. PLA and Ecoflex^®^ are degradable, however, their degradation is so slow on the surface of the soil that they can retain their shape for five months. Therefore, the sheet was maintained by PLA and Ecoflex^®^ during the field test and the voids shown in Figure 12d appeared as traces of the modified starch.

**Figure 14.**
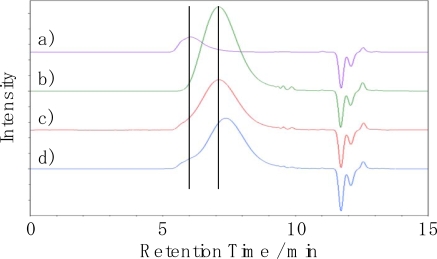
GPC charts of (a) PLA; (b) Ecoflex**^®^**; (c) unused MS-3,6,1; (d) used MS-3,6,1.

The molecular weight was measured by GPC for the evaluation of degradation. The GPC charts are shown in Figure 14. Modified starch could not be dissolved in the chloroform used as the eluent, therefore the GPC charts represent only the peaks of PLA and Ecoflex^®^. A refractometer was used as the GPC detector. The peak intensity of PLA, which has a refractive index similar to chloroform and lower than Ecoflex^®^, even if the concentration of each sample was almost the same (1 w/v%). The chart of the unused MS-3,6,1 shown in Figure 14c indicates that the peaks of PLA and Ecoflex^®^ were combined in one peak. When comparing the unused and used MS-3,6,1, the molecular weight of Ecoflex^®^ decreased, however, that of PLA scarcely decreased. It shows that Ecoflex^®^ degraded after the modified starch and PLA had not yet degraded.

These results show that modified starch degraded almost immediately. Although Ecoflex^®^ also degraded, the shape of the sheets was maintained and the degradation is just decreasing the molecular weight and reducing the strength. Thus, the disappearance of the modified starch formed the voids in the sheet which kept the same shape due to the PLA and Ecoflex^®^ before mulching. The voids formed with time are not the only trace of degradation, but act as a controller of the moisture regain by the soil for the growth of the mandarin orange. It shows that MS-3,6,1 can change the some properties due to the degradability of the constituent materials with time.

### 2.5. Biomass Carbon Ratios of Mulch Sheets

PLA and modified starch are derived from biobased feed stocks. Determination of the biomass carbon ratio is an important issue in the promotion of products fabricated using biorenewable resources. The biomass carbon ratio was determined from the ^14^C concentration measured using AMS based on the ASTM D6866 standard method. This measurement method was published in 2004; however, the compatibility and effectiveness of this method for polymer composites has not yet been reported. The biomass carbon ratios are determined from the mulch sheets is oxidized to form CO_2_ with CuO in a quartz tube at 500–850 °C. The CO_2_ is reduced to graphite with Fe, as explained in the experimental section. The reference materials were measured for the obtained graphite using AMS. The biomass carbon ratios are calculated as follows:

(1) $$\Delta^{14}\text{C}=\left[{(}^{14}\text{As}{-}^{14}\text{Ar}){/}^{14}\text{Ar}\right]\times 1000\;\;\left(‰\right)$$

(2) $$\text{pMC}{=}^{14}\text{C}/10+100\;(\%)$$

(3) Biomass carbon ratio = 0.93 × pMC (%)

The biomass carbon ratio of the unused and used MS-3,6,1 mulch sheets is shown in Table 5. The biomass carbon ratio is the integrated carbon ratio of PLA and modified starch because Ecoflex^®^ is an oil-based material. The biomass carbon ratio of MS-3,6,1 does not match a theoretical value which is calculated from compounding ratio. Polymer composites often segregate each material in molding, then the actual material ratio mismatches the theoretical compounding ratio. Therefore, the actual biomass carbon ratio of MS-3,6,1 was measured by elemental analysis and ^1^H-NMR, and the ratio coincided with the biomass carbon ratio of MS-3,6,1. The biomass carbon ratio of the used MS-3,6,1 decreased. It seems that the decreasing biomass carbon represents the lost modified starch and the value coincides to part of the modified starch ratio. This is also the evidence for the degradation of the modified starch, which derived from biobased feed stocks, because PLA did not degrade from the results of the GPC analysis. This result means that the evaluation method of the biomass carbon ratio is applicable to the degraded material and can be used as an evaluation method of degradability.

## 3. Experimental Section

### 3.1. Materials and Molding

Poly(lactic acid) (Mn = 1.0 × 10^5^, Mw = 2.0 × 10^5^) as H-100 made from biomass feedstock was purchased from Mitsui Chemical, Inc. Ecoflex^®^ (Mn = 2.0 × 10^4^, Mw = 4.8 × 10^4^) was purchased from BASF. Modified starch made from biomass feed stock was purchased from Yamato Co., Ltd. All materials were molded without further drying. The sheet was molded by an IKEGAI FS30 (L/D = 25) single screw extruder equipped with a 120 mm die for inflation molding. The materials went through into the hopper after dry blending. The inflation sheet was rolled up and cut one side to use as a single layered sheet.

### 3.2. Field Tests and Evaluation of Fruit

The field tests were carried out at Wakayama Research Center of Agriculture, Forestry and Fisheries, Fruit Tree Experiment Station. The tested field was a flat field in 2004 and a terraced field in 2005. Taguchi-Wase (*Citrus unshiu Marcovitch)*, an early ripening type of Satsuma mandarin, was used as the mandarin trees. The sheets with 4 × 4 m mulched around the tree until harvest day. The pF values was measured with a ceramic soil water meter (SPAD pF-33; Daiki Rika Kogyo) which installed under 150 mm within 300 mm from the tree. The temperature was measured with drip-proof digital thermometer (MT-804, Mother Tool) which installed under 50 mm within 100 mm from the tree. Mandarin oranges were harvested (30 fruits from four trees) and *a* values were measured with a spectrophotometer (CE-3100, Macbeth). The harvested fruit was crushed after peeling the fruit for measuring of Brix degree and acid value. The Brix degree was measured with an abbe refractometer (DR-A1, ATAGO). The acid value was titrated with 0.1 mol/L sodium hydroxide and converted into the concentration of citric acid as follows:

(4) Acid value (%) = 0.064 (g)/sample (g) × 100

### 3.3. Measurements of Sheets

The diffuse transmittance and reflectance measurements were measured with a UV-Vis spectrometer (UV-2550, SHIMADZU) equipped with an integration sphere (ISR-240A, SHIMADZU). Gas permeability was according to ISO 15105-1 “Plastics - Film and sheeting - Determination of gas-transmission rate - Part 1: The Differential-pressure methods” and measured with a gas permeation measurement apparatus (10XAWT, GTR-Tech) equipped with a gas chromatography (G2700T, Yanaco). The mechanical properties of mulch sheets were measured with a universal material testing machine (Model 5569, INSTRON) at a cross-head speed of 100 mm/min. The materials were cut to 150 × 10 mm test pieces for tension strength test, and 150 × 50 mm test piece which slit down the center for the tearing strength test. Strength at break was calculated based on the dynamic tensile diagrams. The morphology was observed with a scanning electron microscopy (JSM-6480LV, JEOL). The materials were mounted on the sample stub and were coated with gold. The IR spectrum was measured with an IR Prestige-21, SHIMADZU infrared spectrometer equipped with a single reflection ATR system (Durasample IR II, SENSIR TECHNOLOGIES). The GPC was performed at 40 °C with a HLC-8320G instrument (TOSOH) equipped with two columns (TSKgel SuperHM-N, TOSOH) at a flow rate of 1 mL min^−1^. Chloroform served as eluent and a differential refractometer as detector. The materials were prepared with 1 w/v% chloroform solution. ^1^H-NMR spectra were recorded on a JNM-ECX-400 nuclear magnetic resonance spectrometer (JEOL) in chloroform using tetramethylsilane as an internal standard. Elemental analysis was measured with a CHN corder (EA1100, CE Instruments).

### 3.4. Biomass Carbon Ratio

The sample preparation and measurements were performed at the Institute of Accelerator Analysis, Ltd. (IAA), Japan. All carbon atoms in the mulch sheet were transferred to graphite carbons through serial oxidation and reduction reactions using a quartz tube and a vacuum manifold system. Graphite was synthesized from CO_2_ which is oxidized from mulch sheet. Pure graphite with oxidized iron was transferred to a sample holder. The measurement of the ratio of the three carbon isotopes (^14^C, ^13^C, and ^12^C) using AMS was performed at the IAA. The AMS measurement procedure is outlined in Figure 2. The carbon in graphite was ionized using a cesium cation beam. The reduced carbon atoms were accelerated using a 3MV tandem accelerator (NEC Pelletron, 9SDH-2). The amount of ^12^C and ^13^C was detected as a current using multi-Faraday cups. 14C atoms were detected using a solid-state detector with a semiconductor absorber. The ratio of ^14^C to ^12^C concentrations (^14^As) for the mulch sheets was calculated from the measured amounts of ^14^C and ^12^C.

The percentage of modern carbon (pMC) for an oil-based carbon is 0%. The pMC for biomass made from the fixation of CO_2_ in the modern atmosphere through photosynthesis is 108–110% on 2002. The measurement of a product’s ^14^As(^14^C/^12^C) content is determined relative to the modern carbon-based oxalic acid radiocarbon [Standard Reference Material (SRM) 4990c, National Institute of Standards and Technology (NIST), USA].

## 4. Conclusions

A mulch sheet with properties that change during mandarin orange fruit growth could be developed by the inflation molding of PLA, Ecoflex^®^ and modified starch. The mulch sheet is an effective tool for the growth of mandarin oranges. Biodegradable mulch sheets are often used, however, the purpose is just for decreasing of waste after use. The mulch sheet consisting of PLA, Ecoflex^®^ and modified starch has other good biodegradable properties. It seemed that PLA and Ecoflex^®^ are hardly biodegraded by the soil after several months, meanwhile, modified starch has the same biodegradability as raw starch and is lost within a few months. We carried out the field test using the mulch sheets based on the growth of mandarin oranges. The quality of the mandarin orange became better by using the mulch sheets. Furthermore, the inclusion of the modified starch in the sheet made it even better. This advantage for the growth of mandarin oranges is derived from the difference in the degradability of the materials. By blending with some materials, which have different degradabilities, the properties can be controlled by time or condition. In this study, as modified starch is most the degradable material in the mulch sheet, the degradation of modified starch formed some voids in the mulch sheet. The degradation of modified starch induced the controlled effect of moisture regain by the soil, and the change was suitable for the growth of mandarin oranges. Finally, because three materials are fully biodegradable, the mulch sheet can degrade to CO_2_ and H_2_O. In addition to the control of the moisture regain by the soil, PLA and modified starch are biobased materials. Therefore, we evaluate the biomass carbon ratios by the ASTM D6866 standard method. It was confirmed that the mulch sheet includes theoretical biomass carbon ratios and the lack of biobased modified starch decreased the biomass carbon ratios after use in the mandarin orange grove. The evaluation method of the biomass carbon ratio is applicable for the degraded material.

## Figures and Tables

**Table 1.** Ratios of polylactic acid(PLA)/Ecoflex**^®^**/modified starch for inflation molding and their moldability.

**Sheet name**	**Composition/wt%**			**Moldability**
	**PLA**	**Ecoflex**	**Modified starch**	
MS-10,0,0	100	0	0	un-molding
MS-3,0,7	30	0	70	un-molding
MS-3,5,2	30	50	20	un-molding
MS-3,7,0	30	70	0	good
MS-3,6,1	30	60	10	good

**Table 2.** Optical properties of sheets and soil. a) soil surface without covering.

**Sheet name**	**Thickness/μm**	**Diffuse transmittance/%**	**Diffuse reflectance/%**
None^a^	-	-	15~20
MS-3,7,0	30	15	30
	90	51	36
MS-3,6,1	30	62	30
	90	51	36

**Table 3.** Gas permeabilities of the mulch sheets.

**Sheet name**	**Thickness/μm**	**Permeated gases**		
		**Oxygen ml/m^2^day atom**	**Nitrogen ml/m^2^day atom**	**Moisture g/m^2^day**
MS-3,7,0	30	2400	620	85
	90	900	170	51
MS-3,6,1	30	2000	540	51
	90	830	230	46

**Table 4.** Quality of mandarin oranges in 2004 and 2005 1).

**Growth year**	**Thickness/μm**	**Sheet name**	**Brix degree/%**	**Acid value/%**	**Peel color/a value**
2004	30	unmulched	9.0 ± 0.3	0.68 ± 0.05	21.7 ± 1.0
		MS-3,7,0	10.3 ± 0.2	0.74 ± 0.09	25.3 ± 1.4
		MS-3,6,1	10.9 ± 0.6	0.74 ± 0.11	25.1 ± 1.2

2005	90	unmulched	10.3 ± 0.3	0.86 ± 0.10	19.8 ± 1.8
		MS-3,7,0	13.6 ± 0.5	1.09 ± 0.04	23.3 ± 0.6
		MS-3,6,1	13.9 ± 0.5	1.10 ± 0.15	25.3 ± 1.2

1) average of 30 fruits harvested from four trees.

**Table 5.** Biomass carbon ratios of mulch sheets calculated from Δ^14^C measured by accelerated mass spectrometry (AMS) based on ASTM D6866.

**Sheet name**	**^14^C1)/‰**	**pMC 1)/%**	**Biomass carbon ratio2)/%**	**Theoretical biomass carbon ratio 3)/%**	**Carbon ratio of modified starch 3)/%**
unused MS-3,6,1	−604.4 ± 1.9	39.56 ± 0.19	36.79 ± 0.18	38.2	9.8
used MS-3,6,1	−651.2 ± 1.7	34.88 ± 0.17	32.44 ± 0.16	-	-

1) Measured by AMS.

2) Calculation based on Equation 3.

3) measured by ^1^H-NMR and elemental analysis.
